# U-shaped relationship between non-high-density lipoprotein cholesterol and cognitive impairment in Chinese middle-aged and elderly: a cross-sectional study

**DOI:** 10.1186/s12889-024-19164-8

**Published:** 2024-06-18

**Authors:** Lei Li, Lingdan Zhuang, Zichen Xu, Luqing Jiang, Ying Zhai, Daoqin Liu, Qiwen Wu

**Affiliations:** 1 Clinical Laboratory, The First Affiliated Hospital of Wannan Medical College, No. 2, West Zheshan Road, Wuhu, Anhui 241001 China; 2 Department of Kidney Medicine, The First Affiliated Hospital of Wannan Medical College, No. 2, West Zheshan Road, Wuhu, Anhui 241001 China

**Keywords:** Non-HDL-C, Cognitive impairment, CHARLS, U-shaped relationship

## Abstract

**Background:**

The relationship between blood lipids and cognitive function has long been a subject of interest, and the association between serum non-high-density lipoprotein cholesterol (non-HDL-C) levels and cognitive impairment remains contentious.

**Methods:**

We utilized data from the 2011 CHARLS national baseline survey, which after screening, included a final sample of 10,982 participants. Cognitive function was assessed using tests of episodic memory and cognitive intactness. We used multiple logistic regression models to estimate the relationship between non-HDL-C and cognitive impairment. Subsequently, utilizing regression analysis results from fully adjusted models, we explored the nonlinear relationship between non-HDL-C as well as cognitive impairment using smooth curve fitting and sought potential inflection points through saturation threshold effect analysis.

**Results:**

The results showed that each unit increase in non-HDL-C levels was associated with a 5.5% reduction in the odds of cognitive impairment (OR = 0.945, 95% CI: 0.897–0.996; *p* < 0.05). When non-HDL-C was used as a categorical variable, the results showed that or each unit increase in non-HDL-C levels, the odds of cognitive impairment were reduced by 14.2%, 20.9%, and 24% in the Q2, Q3, and Q4 groups, respectively, compared with Q1. In addition, in the fully adjusted model, analysis of the potential nonlinear relationship by smoothed curve fitting and saturation threshold effects revealed a U-shaped relationship between non-HDL-C and the risk of cognitive impairment, with an inflection point of 4.83. Before the inflection point, each unit increase in non-HDL-C levels was associated with a 12.3% decrease in the odds of cognitive impairment. After the tipping point, each unit increase in non-HDL-C levels was associated with an 18.8% increase in the odds of cognitive impairment (All *p* < 0.05).

**Conclusion:**

There exists a U-shaped relationship between non-HDL-C and the risk of cognitive impairment in Chinese middle-aged and elderly individuals, with statistical significance on both sides of the turning points. This suggests that both lower and higher levels of serum non-high-density lipoprotein cholesterol increase the risk of cognitive impairment in middle-aged and elderly individuals.

**Supplementary Information:**

The online version contains supplementary material available at 10.1186/s12889-024-19164-8.

## Introduction

Cognitive impairment is a pathological process that represents the clinical precursor stage between healthy aging and dementia [1]. According to the 2023 World Alzheimer’s Disease Report, the global number of dementia patients is projected to increase from 55 million in 2019 to 139 million by 2050. Associated costs related to dementia are also expected to more than double. Furthermore, according to the 2022 China Alzheimer’s Disease Report, the incidence and mortality rates of Alzheimer’s disease are rapidly rising, making it the fifth leading cause of death among urban and rural residents in China. This places a substantial economic burden on individuals, families, and society as a whole. To date, no specific etiological treatment methods have been discovered for dementia, highlighting the critical importance of early identification of cognitive impairment [2]. Researchers and clinicians widely acknowledge that cognitive impairment represents a potential “window” during which interventions can be undertaken to delay the progression of dementia [3]. Diabetes, hypertension, smoking, and various other risk factors are closely associated with cognitive impairment [4–7]. Furthermore, lipid levels are considered a significant risk factor for cognitive dysfunction. A substantial body of prospective and cross-sectional observational studies has indicated associations between cognitive impairment and abnormal levels of blood lipids or lipoproteins, including total cholesterol (TC), triglycerides (TG), low-density lipoprotein cholesterol (LDL-C), and high-density lipoprotein cholesterol (HDL-C) [8–13].

Non-high-density lipoprotein cholesterol (non-HDL-C) is a cholesterol content marker used to assess the risk of atherosclerosis, including LDL-C, intermediate-density lipoprotein cholesterol (IDL-C), very low-density lipoprotein cholesterol (VLDL-C), chylomicron remnant cholesterol, and lipoprotein A (Lp(a)) [14–16]. Compared to LDL-C, non-HDL-C is more straightforward and exhibits stronger predictive capabilities. In the assessment of atherosclerotic cardiovascular disease (ASCVD) risk, non-HDL-C performs on par with LDL-C, and in patients with mild to moderate hypertriglyceridemia, it outperforms LDL-C [17]. Research indicates that serum non-HDL-C levels are higher in individuals with cognitive impairment compared to those with normal cognitive function, and cognitive function scores are inversely correlated with non-HDL-C levels [18]. Additionally, studies utilizing the NHANES database have discovered a positive and non-linear correlation between serum non-HDL-C levels and cognitive function in older Americans [19].

The relationship between lipids and cognitive function remains a subject of debate, and there has been limited investigation into the linear or nonlinear association between serum non-HDL-C and cognitive abilities in middle-aged and elderly Chinese individuals. Therefore, the present study aims to explore the correlation between serum non-HDL-C and cognitive abilities in this population.

## Materials and methods

### Study population

The data for this study were sourced from the China Health and Retirement Longitudinal Study (CHARLS). CHARLS is a large-scale interdisciplinary survey project led by the National School of Development at Peking University, jointly executed by the China Social Science Survey Center at Peking University and the Youth League Committee of Peking University. The project is supported by the National Natural Science Foundation of China and aims to collect high-quality microdata representing the family and individual circumstances of Chinese adults aged 45 and above. These data are employed to conduct in-depth analyses of China’s aging population, foster interdisciplinary research, and provide scientific evidence for policy formulation. CHARLS conducts regular follow-up surveys every 2 to 3 years, with blood sample collection carried out only in 2011 and 2015.

**Fig. 1:**
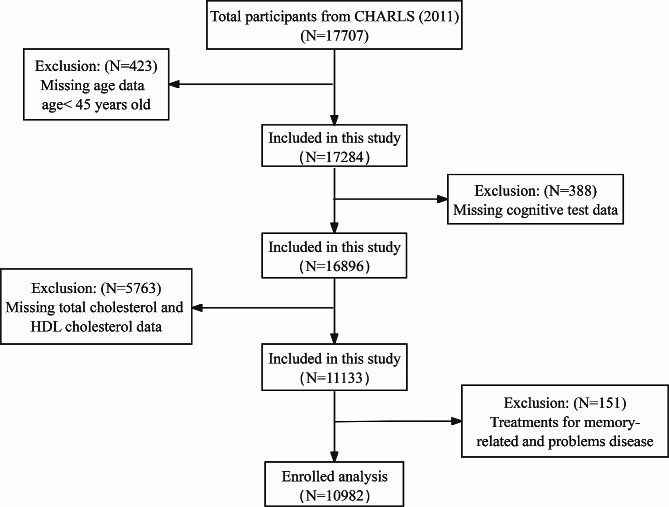
Participant selection process flowchart

The baseline survey (Wave 1) took place in 2011, with a total of 17,707 participants. During the data screening process, participants under the age of 45, those lacking cognitive or cholesterol data, individuals taking memory-related psychiatric medications, and those missing data on total cholesterol and high-density cholesterol were excluded. Ultimately, the study included 10,982 participants. Further details regarding the selection of the study population are provided in Fig. 1.

All participants provided informed consent before participating in the study, and ethical approval for data collection in CHARLS was obtained from the Biomedical Ethics Review Committee of Peking University (IRB00001052-11015). Only individuals who provided written informed consent were included in the study.

### Cognitive function assessment

Cognitive function in CHARLS is assessed using MMSE, a classic tool that evaluates cognitive function through tests of episodic memory and mental intactness. The assessment of episodic memory encompassed two aspects: immediate word recalls and delayed word recall. In immediate word recall, participants were presented with a list of ten words, after which they were asked to recall as many of these words as possible. In delayed word recall, participants were required to repeat the same words again after a 5-minute interval. Each correctly recalled word contributed 1 point, resulting in a total episodic memory score ranging from 0 to 20.

The evaluation of mental intactness included assessments of numerical ability, time orientation ability, and figure drawing ability. The numerical ability test involved participants starting from 100 and subtracting 7 consecutively, repeating this process five times. Time orientation assessment required participants to provide the survey date (including month, day, year), the day of the week, and the season. In the figure drawing task, participants were shown an image composed of two overlapping pentagons and asked to replicate it. Similar to episodic memory, each correct answer or successfully reproduced image scored 1 point, with a maximum mental intactness score of 11. The composite scores from episodic memory and mental intactness constituted the overall cognitive score, which ranged from 0 to 31 points. Based on prior research, cognitive impairment is typically defined as an overall cognitive score below 11 points [20, 21].

### Definition of non-HDL-C

In the national survey, blood samples from participants were collected from the Chinese Center for Disease Control and Prevention, all of whom underwent rigorous training by staff. After collection, blood samples were transported to local laboratories and stored at 4 ℃. After the venous blood was separated into plasma and buffy coat, the plasma was then stored in three 0.5 mL cryovials and the buffy coat in a separate cryovial. These cryovials were then immediately stored frozen at − 20 ℃ and transported to the Chinese CDC in Beijing within 2 weeks where they were placed in a deep freezer and stored at − 80 ℃ until assay at CMU laboratory. Calculate non-HDL-C levels by subtracting HDL-C from TC.

### Covariates

Based on previous research, we will consider demographic and health-related factors as covariates in our study analysis. Demographic covariates include gender (male and female), age, education (categorized as illiterate, primary school, and middle school and above), marital status (married and cohabiting, and others, including divorced, widowed, unmarried, and separated). Health-related covariates encompass Body Mass Index (BMI), hypertension, diabetes, dyslipidemia, exercise, socializing, smoking, drinking, antihypertensive therapy, hypoglycemic therapy, lipid medication, and depression.

BMI is calculated as weight (in kilograms) divided by height (in meters) squared, with units in kg/m². Hypertension is defined as blood pressure exceeding 140/90 mmHg (based on the average of three measurements) or self-reported hypertension by participants. Diabetes is defined as self-reported diabetes, use of antidiabetic medication, or fasting blood glucose ≥ 7.0 mmol/L. Dyslipidemia is defined as self-reported lipid abnormalities or the use of related medications. Exercise is defined as engaging in physical labor at least once per week. Socializing is defined as participation in one of ten activities in the previous month, such as interacting with friends, playing mahjong, chess, card games, or going to community clubs. As long as a participant engages in one of these activities, participants are considered to have participated in social activities. Smoking is defined as current smoking, never smoking, or former smoking. Drinking is defined as drinking alcohol at least once per month. CHARLS uses the 10-item Center for Epidemiologic Studies Depression Scale (CESD-10) to assess depression in participants, which has been demonstrated as an effective, reliable, and practical tool for mental health assessment [22, 23]. Based on previous research, participants scoring 10 or above on this scale are defined as having depressive symptoms [23, 24].

### Statistical analysis

Quantitative data are presented as mean ± standard deviation (SD), while qualitative data are expressed as numbers (percentages). Differences among the various quartile groups of non-HDL-C were analyzed using the chi-square test or Kruskal-Wallis H test. We employed a multiple regression model to assess the correlation between non-HDL-C and the binary categorical variable of cognitive impairment. Three distinct models were established: Non-adjusted; Model I, adjusted for gender and age; and Model II, with comprehensive adjustments, including gender, age, BMI, education, marital status, hypertension, diabetes, dyslipidemia, depression, antihypertensive therapy, hypoglycemic therapy, lipid medication, smoking, drinking, exercise, and socializing, among other factors. Subsequently, based on the adjusted variables of Model II, we utilized smoothed curve fitting to explore potential nonlinear relationships that may exist between non-HDL-C and cognitive impairment. Additionally, saturation threshold effect analysis was employed to identify potential inflection points. Furthermore, subgroup analyses were conducted based on factors such as gender, age, BMI, education, marital status, depression, smoking, drinking, exercise, and socializing, to investigate whether these factors influenced the relationship between non-HDL-C and the risk of cognitive impairment. To support the consistency of our results, we also conducted corresponding statistical analyses on the cognitive scores.

## Results

### Baseline characteristics of participants based on non-HDL-C quartiles

A total of 10,982 participants were enrolled in this study. Participants were categorized into four groups based on their non-HDL-C quartiles: Q1 (≤ 2.99 mmol/L), Q2 (2.99 to ≤ 3.59 mmol/L), Q3 (3.59 to ≤ 4.27 mmol/L), and Q4 (> 4.27 mmol/L), with units in mmol/L. The baseline characteristics are presented in Table 1.

**Table 1:** Based on the demographic and clinical characteristics of non-HDL cholesterol levels

	Non-HDL cholesterol categories				
	Q1(< 2.99 mmol/L)	Q2(2.99–3.59 mmol/L)	Q3(3.59–4.27 mmol/L)	Q4(4.27–15.92 mmol/L)	*p*-value
N	2745	2746	2745	2746	
Age (years)	59.35 ± 9.99	59.37 ± 9.65	59.32 ± 9.50	59.71 ± 9.20	0.103
Age group					0.617
45–60	1513 (55.12%)	1503 (54.73%)	1526 (55.59%)	1479 (53.86%)	
≧ 60	1232 (44.88%)	1243 (45.27%)	1219 (44.41%)	1267 (46.14%)	
Gender					< 0.001***
Male	1547 (56.40%)	1351 (49.20%)	1233 (44.97%)	1081 (39.41%)	
Female	1196 (43.60%)	1395 (50.80%)	1509 (55.03%)	1662 (60.59%)	
Education					0.032*
Illiterate	734 (26.76%)	761 (27.73%)	773 (28.19%)	843 (30.73%)	
Primary school	1143 (41.67%)	1091 (39.76%)	1123 (40.96%)	1062 (38.72%)	
Middle school and above	866 (31.57%)	892 (32.51%)	846 (30.85%)	838 (30.55%)	
Marital status					0.987
Married and cohabiting	2426 (88.38%)	2420 (88.13%)	2418 (88.09%)	2423 (88.24%)	
Others	319 (11.62%)	326 (11.87%)	327 (11.91%)	323 (11.76%)	
Hypertension					< 0.001***
No	1850 (67.59%)	1703 (62.18%)	1599 (58.53%)	1439 (52.50%)	
Yes	887 (32.41%)	1036 (37.82%)	1133 (41.47%)	1302 (47.50%)	
Diabetes					< 0.001***
No	2398 (87.36%)	2347 (85.47%)	2321 (84.55%)	2093 (76.22%)	
Yes	347 (12.64%)	399 (14.53%)	424 (15.45%)	653 (23.78%)	
Dyslipidemia					< 0.001***
No	2198 (81.41%)	2057 (76.64%)	1978 (73.78%)	1778 (65.88%)	
Yes	502 (18.59%)	627 (23.36%)	703 (26.22%)	921 (34.12%)	
Depression					0.017*
No	1449 (52.90%)	1540 (56.20%)	1517 (55.55%)	1450 (52.86%)	
Yes	1290 (47.10%)	1200 (43.80%)	1214 (44.45%)	1293 (47.14%)	
Antihypertensive therapy					< 0.001***
No	2331 (84.92%)	2229 (81.17%)	2177 (79.31%)	2021 (73.60%)	
Yes	414 (15.08%)	517 (18.83%)	568 (20.69%)	725 (26.40%)	
Hypoglycemic therapy					< 0.001***
No	2665 (97.09%)	2644 (96.29%)	2631 (95.85%)	2597 (94.57%)	
Yes	80 (2.91%)	102 (3.71%)	114 (4.15%)	149 (5.43%)	
Lipid medication					< 0.001***
No	2671 (97.30%)	2633 (95.88%)	2598 (94.65%)	2525 (91.95%)	
Yes	74 (2.70%)	113 (4.12%)	147 (5.35%)	221 (8.05%)	
Smoking					< 0.001***
No	1787 (65.10%)	1933 (70.39%)	1981 (72.17%)	2108 (76.77%)	
Yes	958 (34.90%)	813 (29.61%)	764 (27.83%)	638 (23.23%)	
Drinking					< 0.001***
No	1686 (61.67%)	1826 (66.72%)	1852 (67.89%)	1935 (70.59%)	
Yes	1048 (38.33%)	911 (33.28%)	876 (32.11%)	806 (29.41%)	
Exercise					0.290
No	1608 (58.58%)	1655 (60.27%)	1611 (58.69%)	1586 (57.76%)	
Yes	1137 (41.42%)	1091 (39.73%)	1134 (41.31%)	1160 (42.24%)	
Socializing					< 0.001***
No	1514 (55.16%)	1399 (50.95%)	1342 (48.89%)	1355 (49.35%)	
Yes	1231 (44.84%)	1347 (49.05%)	1403 (51.11%)	1391 (50.65%)	
Cognitive impairment					0.003**
No	1682 (61.28%)	1752 (63.80%)	1800 (65.57%)	1795 (65.37%)	
Yes	1063 (38.72%)	994 (36.20%)	945 (34.43%)	951 (34.63%)	
Cognitive score	12.87 ± 6.65	13.14 ± 6.71	13.33 ± 6.54	13.30 ± 6.64	0.027*
SBP (mmHg)	127.49 ± 21.02	130.09 ± 21.40	132.18 ± 21.98	134.29 ± 21.80	< 0.001***
DBP (mmHg)	73.92 ± 12.19	75.470 ± 12.18	76.53 ± 11.98	77.80 ± 12.14	< 0.001***
BMI (kg/m^2)^	22.32 ± 3.60	23.22 ± 3.80	23.89 ± 4.02	24.63 ± 3.89	< 0.001***
WC (cm)	80.85 ± 12.32	83.35 ± 12.55	85.49 ± 12.03	87.76 ± 12.51	< 0.001***
Triglycerides (mmol/L)	1.00 ± 0.52	1.29 ± 0.77	1.56 ± 0.89	2.29 ± 1.95	< 0.001***
Total Cholesterol (mmol/L)	3.96 ± 0.54	4.65 ± 0.43	5.19 ± 0.41	6.24 ± 0.80	< 0.001***
HDL Cholesterol (mmol/L)	1.43 ± 0.42	1.34 ± 0.40	1.28 ± 0.37	1.22 ± 0.36	< 0.001***
LDL Cholesterol (mmol/L)	2.14 ± 0.43	2.752 ± 0.43	3.22 ± 0.52	3.91 ± 1.01	< 0.001***
Non-HDL Cholesterol (mmol/L)	2.53 ± 0.36	3.30 ± 0.17	3.91 ± 0.19	5.02 ± 0.77	< 0.001***

SBP, systolic blood pressure; DBP, diastolic biood pressure; BMI, body mass index; WC, waist circumference. *p < 0.05, **p < 0.01, ***p < 0.001

Compared to the other groups, participants in the highest quartile (Q4) had a higher proportion of females, individuals with hypertension, lipid abnormalities, diabetes, limited social activities, and depression. Conversely, the proportion of males, smokers, and alcohol consumers was lower in this group. Additionally, participants with higher non-HDL-C levels tended to have elevated BMI, WC, TG, TC, and LDL-C, while their HDL-C levels were lower. Furthermore, in comparison to the lowest quartile (Q1), participants in the other quartiles exhibited higher cognitive scores and a lower proportion of participants with cognitive impairment.

### Multivariate regression analysis of non-HDL-C with cognitive impairment

Table 2 illustrates the multivariate linear regression analysis of Non-HDL-C with cognitive impairment. After full adjustment, participants with high Non-HDL-C levels exhibited better cognitive function. The odds ratio suggests that for every unit increase in non-HDL-C levels, there is a 5.5% decrease in the odds of cognitive impairment. (OR = 0.945, 95% CI: 0.897–0.996; *p* < 0.05). We stratified Non-HDL-C into four quartiles, and when Non-HDL-C was used as a categorical variable, the results demonstrated that the risk of cognitive impairment was lower in the other three quartiles compared to participants in the first quartile of Non-HDL-C. The respective ORs for the three quartiles were 0.858 (95% CI, 0.744–0.990, *p* < 0.05), 0.791 (95% CI, 0.684–0.915, *p* < 0.001), and 0.760 (95% CI, 0.655–0.882, *p* < 0.001). These values indicate that for each unit increase in non-HDL-C levels, the odds of cognitive impairment were reduced by 14.2%, 20.9%, and 24% in the Q2, Q3, and Q4 groups, respectively, compared with Q1.

**Table 2:** Association between non-HDL-C and cognitive impairment

Exposure	OR (95%CI), *p*		
	Non-adjusted	Adjust I	Adjust II
Non-HDL-C	0.957 (0.921, 0.995) 0.03*	0.906 (0.869, 0.945) < 0.001***	0.945 (0.897, 0.996) 0.03*
Non-HDL-C quartile			
Q1	Reference	Reference	Reference
Q2	0.898 (0.805, 1.001) 0.05	0.844 (0.752, 0.946) < 0.01**	0.858 (0.744, 0.990) 0.04*
Q3	0.831 (0.744, 0.927) < 0.001***	0.752 (0.670, 0.844) < 0.001***	0.791 (0.684, 0.915) 0.002**
Q4	0.838 (0.751, 0.936) 0.002**	0.710 (0.632, 0.798) < 0.001***	0.760 (0.655, 0.882) < 0.001***
*p* for trend	< 0.001***	< 0.001***	< 0.001***

Non-adjusted

Model I: Adjusted for age, gender

Model II: Adjusted for age, gender, BMI, education, marital status, hypertension, diabetes, dyslipidemia, depression, antihypertensive therapy, hypoglycemic therapy, lipid medication, smoking, drinking, exercise, and socializing

OR: Odds Ratio; 95%CI: 95% confidence interval

*p < 0.05, **p < 0.01, ***p < 0.001

### Nonlinear relationship between non-HDL-C and risk of cognitive impairment

**Fig. 2:**
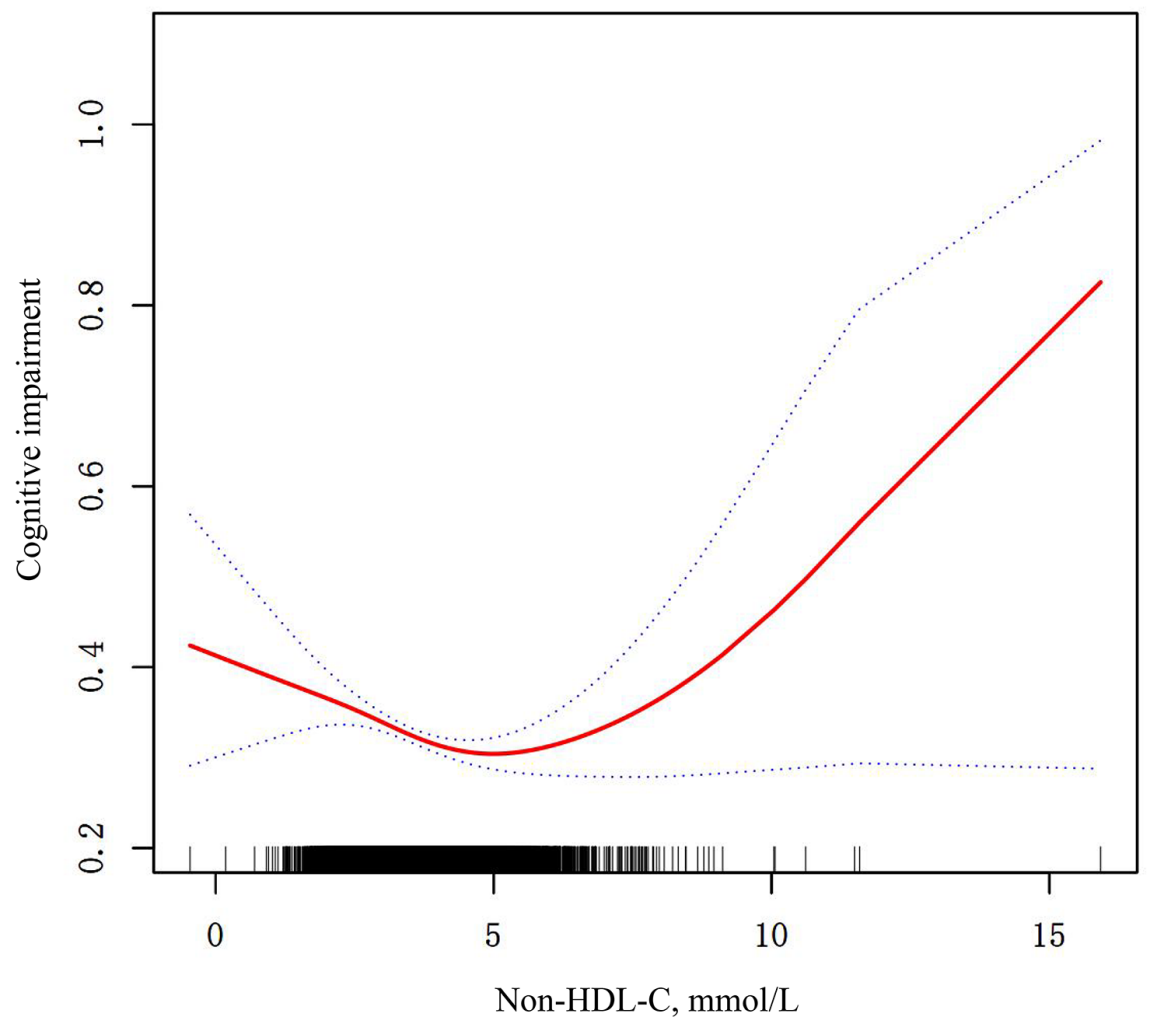
The smoothing curve fitting was used to assess the nonlinear relationship between non-HDL-C and cognitive impairment

We employed smoothed curve fitting to investigate the nonlinear relationship between Non-HDL-C and the risk of cognitive impairment. Figure 2 illustrates that, in the fully adjusted model, there exists a U-shaped correlation between Non-HDL-C and the risk of cognitive impairment. Through saturation threshold effect analysis (Table 3), the inflection point was determined to be 4.83. Before the inflection point, there is a negative association between Non-HDL-C and the risk of cognitive impairment, whereas after the inflection point, there is a positive association. The results showed that before the inflection point, each unit increase in non-HDL-C levels was associated with a 12.3% decrease in the odds of cognitive impairment. After the tipping point, each unit increase in non-HDL-C levels was associated with an 18.8% increase in the odds of cognitive impairment. The respective odds ratios (95% CI) were 0.877 (0.819–0.939, *p* < 0.001) and 1.188 (1.034–1.366, *p* < 0.05).

**Table 3:** Threshold effect analysis of non-HDL cholesterol on cognitive impairment based on model II

Outcome	OR (95%CI), *p*
	cognitive impairment
model I	
Fitting by the standard linear model	0.945 (0.897, 0.996) 0.03*
model II	
Inflection point	4.83
< Inflection point	0.877 (0.819, 0.939) < 0.001***
> Inflection point	1.188 (1.034, 1.366) 0.02*
Log likelihood ratio	< 0.001***

*p < 0.05, **p < 0.01, ***p < 0.001

OR: Odds Ratio; 95%CI: 95% confidence interval

To further validate the robustness of our findings, we included MMSE scores in our analysis. The results obtained with MMSE scores were consistent with those observed for cognitive impairment, demonstrating the reliability of our results. Detailed data and analyses regarding the relationship between non-HDL-C and MMSE scores are provided in the supplementary material. (See Additional file 1 for relevant data)

### Subgroup analysis

**Fig. 3:**
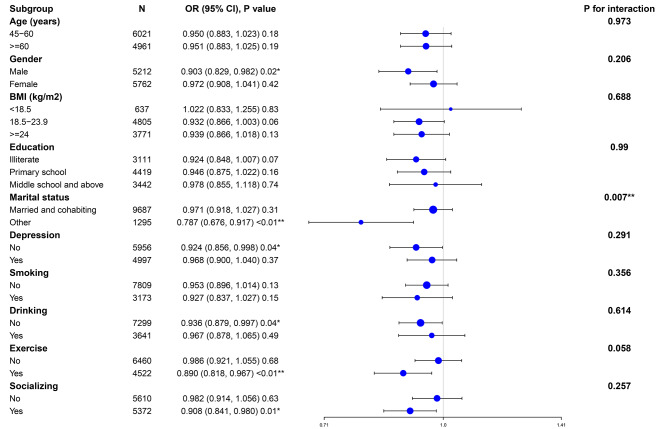
The effect size of non-HDL-C on cognitive impairment in the subgroup. Notes: adjusted for age, gender, BMI, education, marital status, Hypertension, Diabetes, Dyslipidemia, Depression, Antihypertensive therapy, Hypoglycemic therapy, Lipid medication, smoking, drinking, Exercise, and Socializing

In subgroup analyses stratified by gender, age, BMI, education, marital status, depression, smoking, drinking, exercise, and socializing, the results remained consistent after adjusting for confounding factors (Fig. 3). Interaction tests revealed that the risk of cognitive impairment was lower in the married and cohabitating group compared to those with other marital statuses (p for interaction = 0.007). Furthermore, we generated smoothed curves for the relationship between Non-HDL-C and the risk of cognitive impairment and cognitive scores within different subgroups, and the primary findings remained stable (see additional file 2).

## Discussion

Based on a cross-sectional study involving 10,982 Chinese middle-aged and older adults, our findings suggest that higher levels of serum non-HDL-C are associated with better cognitive performance within a certain range. Further analysis using smoothed curve fitting revealed a U-shaped correlation between serum non-HDL-C levels and the risk of cognitive impairment. Through the detection of saturation threshold effects, we identified a turning point at 4.83. Specifically, prior to this turning point, the risk of cognitive impairment significantly decreased with increasing serum non-HDL-C levels (*p* < 0.001). Conversely, after the turning point, the risk of cognitive impairment increased significantly with higher serum non-HDL-C levels (*p* < 0.05). Both sides of this turning point exhibited statistically significant differences.

The connection between blood lipids and cognitive ability is a topic of considerable complexity and remains not entirely elucidated. In previous research [25–27], lipid abnormalities have long been recognized as a significant risk factor for cardiovascular diseases; however, the relationship between plasma lipids and cognitive function continues to be a subject of debate. A retrospective cross-sectional study [18] identified that elevated serum non-HDL-C levels might significantly increase the risk of cognitive impairment following ischemic stroke. The study findings indicate a negative correlation between Mini-Mental State Exam (MMSE) and Montreal Cognitive Assessment (MoCA) scores and non-HDL-C levels. Importantly, after adjusting for confounding factors, non-HDL-C levels were independently associated with the presence of cognitive impairment.

In contrast, Lv et al. [28] conducted a cross-sectional study that included 2,000 elderly individuals aged 65 and older. They found significant positive linear associations between TC, TG, LDL-C, HDL-C, and MMSE scores. For each 1 mmol/L increase in TC, TG, LDL-C, and HDL-C, after adjusting for covariates, MMSE scores increased significantly by 0.67, 0.56, 0.52, and 1.25 points, respectively. When cholesterol measurements were treated as continuous variables, every 1 mmol/L increase in TC, TG, and LDL-C was associated with a decreased risk of cognitive impairment, with corresponding adjusted odds ratios of 0.78 (0.71–0.86), 0.67 (0.56–0.80), and 0.80 (0.72–0.89). Furthermore, another longitudinal study conducted in China also discovered that as non-HDL-C levels increased from low to high, the risk of declining cognitive abilities significantly decreased [20].

We grouped non-HDL-C according to quartiles, and when non-HDL-C was used as a categorical variable, the results showed that participants in the other three quartiles had a lower risk of cognitive impairment compared to those in the first quartile of non-HDL-C, which appeared to present a negative correlation. We obtained intriguing results when we employed smoothed curve fitting to investigate the possible nonlinear relationship between the two variables. We found that the relationship between non-HDL-C and the risk of developing cognitive impairment, on the other hand, showed a U-shaped nonlinear relationship. After reviewing the data, we found that when using non-HDL-C as a categorical variable, its Q4 (> 4.27 mmol/L) cutoff was to the left of the inflection point of the smoothed curve. When using non-HDL cholesterol as a categorical variable to explore the relationship with the risk of cognitive impairment, it may be that the use of quartile groupings is not sufficiently precise because there were few participants with high levels of non-HDL cholesterol. Thus, the direct effect of excessively high levels of non-HDL cholesterol on the elevated risk of developing cognitive impairment may be attenuated when constructing regression equations.

To date, there has been ongoing debate regarding the association between blood lipids and cognitive abilities. Zhang et al. [19] examined the nonlinear relationship between non-HDL-C and cognitive function in American elderly individuals using the NHANES database. They discovered a positive and nonlinear correlation between serum non-HDL-C levels and cognitive function in American elderly individuals. This suggests that maintaining appropriate serum cholesterol levels may contribute to preserving cognitive health in the elderly. In a community-based prospective cohort study, Marcum et al. [29] found an overall significant association between higher Alzheimer’s disease (AD) risk and non-HDL-C levels among individuals aged 60–69 and 70–79. This revealed that there might be a potential U-shaped relationship, indicating higher AD risk at both low and high non-HDL-C levels. Our research findings also support this perspective, as we observed a U-shaped correlation between serum non-HDL-C levels and the risk of cognitive impairment, consistent with previous research results. Furthermore, a population-based registry study involving 118,160 participants who were not undergoing statin treatment found an association between higher lipoprotein levels and lower mortality rates [30]. This suggests that high lipoprotein levels may not necessarily be detrimental in the general population. Taken together, these study results collectively suggest that within a certain range, serum non-HDL-C may have a protective effect on an individual’s cognitive function. It appears that this protective effect differs from its harmful mechanisms in the context of cardiovascular diseases, emphasizing the need for further research to gain a deeper understanding of this relationship.

Our main finding is the existence of a U-shaped non-linear relationship between non-HDL-C levels and the risk of cognitive impairment. Although the exact mechanism between blood lipids and cognitive function remains elusive, this U-shaped relationship has biological plausibility. Firstly, approximately one-quarter of the body’s cholesterol is concentrated in the brain, with lipid components including phospholipids, omega-3, and cholesterol itself constituting nearly half of the brain’s weight [20]. Cholesterol plays a crucial role in the nervous system, serving as a fundamental component of metabolism and cell membranes [31]. It plays a vital role in the maturation and function of neurons, facilitating the assembly of numerous receptors and regulatory molecules, and serving as a platform for intracellular signal transduction. In the brain, cholesterol plays a pivotal role in neurotrophic factor signaling, promoting the formation of lipid rafts, serving as a fundamental component of myelin phospholipids, and being a critical component of synaptic vesicles, thereby maintaining synaptic activity [32]. Research by Mauch et al. [33] has shown that cholesterol is an essential component of myelin phospholipids and synaptic vesicles, with the ability of central nervous system neurons to form synapses being limited by the availability of cholesterol. Cholesterol plays an indispensable role in synaptic plasticity and promoting the processes of learning and memory in the brain.

Secondly, another factor is that cholesterol stores a large amount of energy, which can be continuously supplied to the brain over the long term, considering the brain as the organ that consumes the most energy in the body [34]. Additionally, extremely low levels of cholesterol may indicate early manifestations of chronic diseases, potentially leading to cognitive impairment, weakness, and Alzheimer’s disease (AD) [29, 35]. In animal experiments, intrathecal injection of cholesterol in mice has been shown to prevent the decline in cognitive abilities [36]. Depletion of cholesterol in the brain is strongly associated with cognitive decline. Therefore, the role of cholesterol in brain protection may differ from its role in cardiovascular diseases.

Although peripheral cholesterol cannot cross the blood-brain barrier to enter the brain, oxidized cholesterol is an exception. Increasing evidence supports the role of oxidized cholesterol as a link between altered cholesterol metabolism in the brain and hypercholesterolemia [37, 38]. Previous studies in rabbits and mice have shown that a cholesterol-rich diet can increase the permeability of the blood-brain barrier while also increasing the accumulation of Aβ in the hippocampus [39]. Furthermore, research indicates that oxidized cholesterol is involved in regulating neuroinflammation, Aβ accumulation, and cell death, strongly supporting its critical role in the pathogenesis of AD [40]. When cholesterol levels rise to a certain extent, they may be associated with more complications, significantly increasing the risk of cognitive impairment. Animal experiments by Marcum et al. [29] have found that a high-cholesterol diet increases the accumulation of Aβ and accelerates AD, suggesting that elevated cholesterol levels may accelerate the onset of AD. Current research indicates that within a certain range, non-HDL-C is associated with better cognitive function, emphasizing the importance of enhancing awareness of scientific cholesterol management concepts. Future research will contribute to a deeper understanding of the complex relationship between lipid levels and cognitive function, as well as exploring more precise management strategies.

This study is a cross-sectional research based on a nationally representative sample from China, which enhances the reliability of the statistical outcomes. Furthermore, after adjusting for numerous confounding factors, we identified a U-shaped relationship and determined the inflection point between serum non-HDL-C levels and the risk of cognitive impairment. However, it is important to note that the study has certain limitations. First, it is a cross-sectional study, and therefore, causality cannot be established between serum non-HDL-C levels and the risk of cognitive impairment. Additionally, our study is confined to the Chinese region, and the generalizability of the results may be constrained by geographical factors. Second, our analysis is based on existing databases, and some data may carry uncertainties in terms of accuracy, which should be considered with caution. Third, our study primarily focused on investigating the impact of the relatively new non-HDL-C marker on the risk of cognitive impairment and did not delve into the separate effects of other lipid components in the serum, which is an area requiring further exploration. Lastly, the confounding factors we considered in the analysis may not encompass all potential influencing factors; therefore, some unaccounted confounding variables may impact the results.

## Conclusion

We conducted a study using the Chinese CHARLS database, which contains a nationally representative sample of middle-aged and elderly individuals in China. Our research findings demonstrate a U-shaped relationship between serum non-HDL-C levels and the risk of cognitive impairment in middle-aged and elderly Chinese individuals, with statistically significant inflection points on both sides. This implies that both lower and higher levels of serum non-HDL-C increase the risk of cognitive impairment in this population. In the future, further research is needed to gain a deeper understanding of the impact of lipid components on the risk of cognitive impairment, providing more effective guidance and strategies for the prevention and management of cognitive diseases.

**Declarations**.

### Electronic supplementary material

Below is the link to the electronic supplementary material.

Supplementary Material 1

Supplementary Material 2

## Data Availability

All data can be downloaded and used on the official CHARLS database website. (https://charls.pku.edu.cn)

## Abbreviations

non-HDL-C: Non-high-density lipoprotein cholesterol
OR: Odds Ratio
95% CI: 95% confidence interval
TC: Total cholesterol
TG: Triglycerides
LDL-C: Low-density lipoprotein cholesterol
HDL-C: High-density lipoprotein cholesterol
IDL-C: Intermediate-density lipoprotein cholesterol
VLDL-C: Very low-density lipoprotein cholesterol
Lp(a): Lipoprotein A
ASCVD: Atherosclerotic cardiovascular disease
CHARLS: China Health and Retirement Longitudinal Study
BMI: Body Mass Index
SD: Standard deviation
AD: Alzheimer’s disease
