# Supplementary material for: U-shaped relationship between non-high-density lipoprotein cholesterol and cognitive impairment in Chinese middle-aged and elderly: a cross-sectional study
Source: BMC Public Health. 2024 Jun 18;24:1624. doi: 10.1186/s12889-024-19164-8 (PMC11186169; doi:10.1186/s12889-024-19164-8)
Supplement: Supplementary file 1 — Supplementary Material 1 [file 12889_2024_19164_MOESM1_ESM.docx]

Table S1: Association between non-HDL-C and MMSE scores

| Exposure | β (95%CI), *p* | | |
| --- | --- | --- | --- |
|  | Non-adjusted | Adjust I | Adjust II |
| Non-HDL-C | 0.128 (0.006, 0.250) 0.04* | 0.255 (0.138, 0.372) <0.001*** | 0.060 (-0.043, 0.163) 0.25 |
| Non-HDL-C quartile |  |  |  |
| Q1 | Reference | Reference | Reference |
| Q2 | 0.272 (-0.079, 0.623) 0.13 | 0.411 (0.078, 0.744) 0.02* | 0.176 (-0.111, 0.463) 0.23 |
| Q3 | 0.467 (0.116, 0.818) 0.01* | 0.676 (0.342, 1.010) <0.001*** | 0.254 (-0.036, 0.544) 0.09 |
| Q4 | 0.433 (0.082, 0.784) 0.02* | 0.830 (0.495, 1.165) <0.001*** | 0.342 (0.047, 0.637) 0.02* |
| *p* for trend | 0.01* | <0.001*** | 0.02* |

Non-adjusted.

Model I: Adjusted for age, gender.

Model II: Adjusted for age, gender, BMI, education, marital status, hypertension, diabetes, dyslipidemia, depression, antihypertensive therapy, hypoglycemic therapy, lipid medication, smoking, drinking, exercise, and socializing.

*p<0.05, **p<0.01, ***p<0.001.

β: Regression coefficients; 95%CI: 95% confidence interval


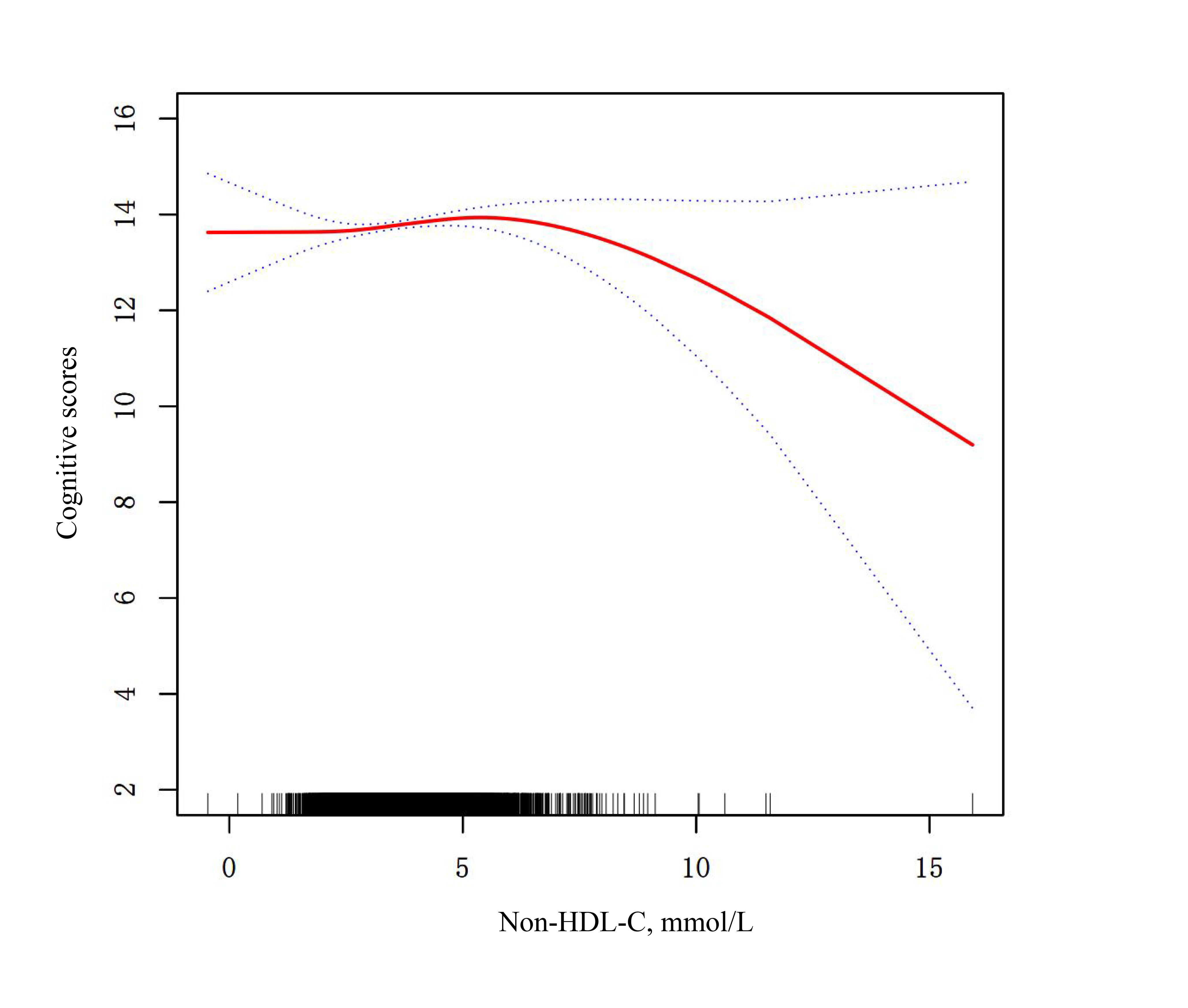


Figure S1: The smoothing curve fitting was used to assess the nonlinear relationship between non-HDL-C and MMSE scores.

Table S2: Threshold effect analysis of non-HDL cholesterol and MMSE scores based on model II.

| Outcome | β (95%CI), *p* |
| --- | --- |
|  | MMSE score |
| model I |  |
| Fitting by the standard linear model | 0.060 (-0.043, 0.163) 0.25 |
| model II |  |
| Inflection point | 5.44 |
| < Inflection point | 0.135 (0.016, 0.255) 0.03* |
| > Inflection point | -0.404 (-0.793, -0.015) 0.04* |
| Log likelihood ratio | 0.02* |

*p<0.05, **p<0.01, ***p<0.001.

β: Regression coefficients; 95%CI: 95% confidence interval
