# Supplementary material for: U-shaped relationship between non-high-density lipoprotein cholesterol and cognitive impairment in Chinese middle-aged and elderly: a cross-sectional study
Source: BMC Public Health. 2024 Jun 18;24:1624. doi: 10.1186/s12889-024-19164-8 (PMC11186169; doi:10.1186/s12889-024-19164-8)

Additional file 2: Subgroup analysis of non-HDL-C versus cognitive impairment and cognitive scores: A.Group according to gender; B.Group according to age; C.Group according to BMI; D.Group according to education; E.Group according to marital status; F.Group according to whether they are depressed or not; G.Group according to whether they smoking or not; H.Group according to whether drinking or not; I.Group according to whether exercise or not; J.Group according to whether socializing or not.


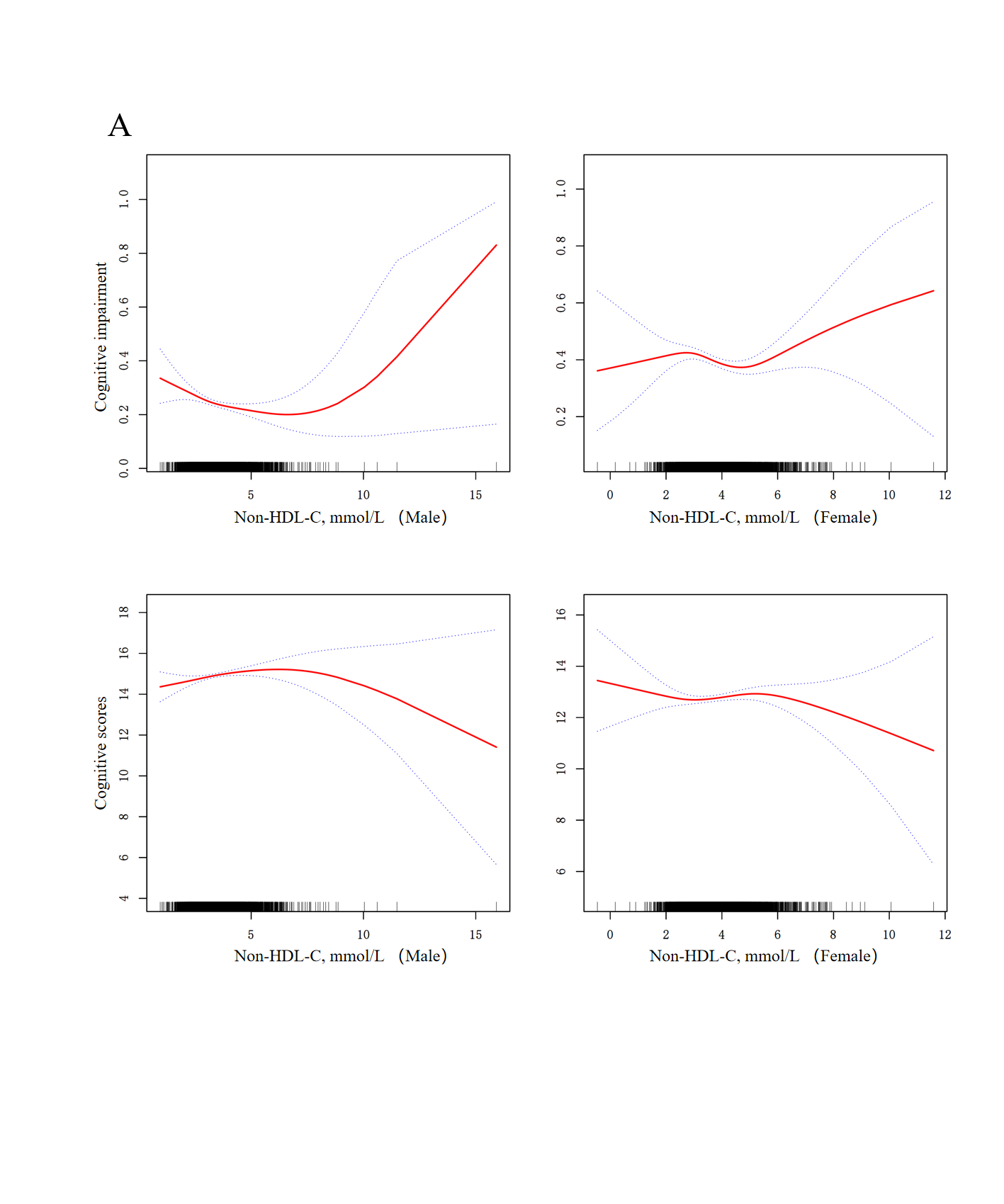


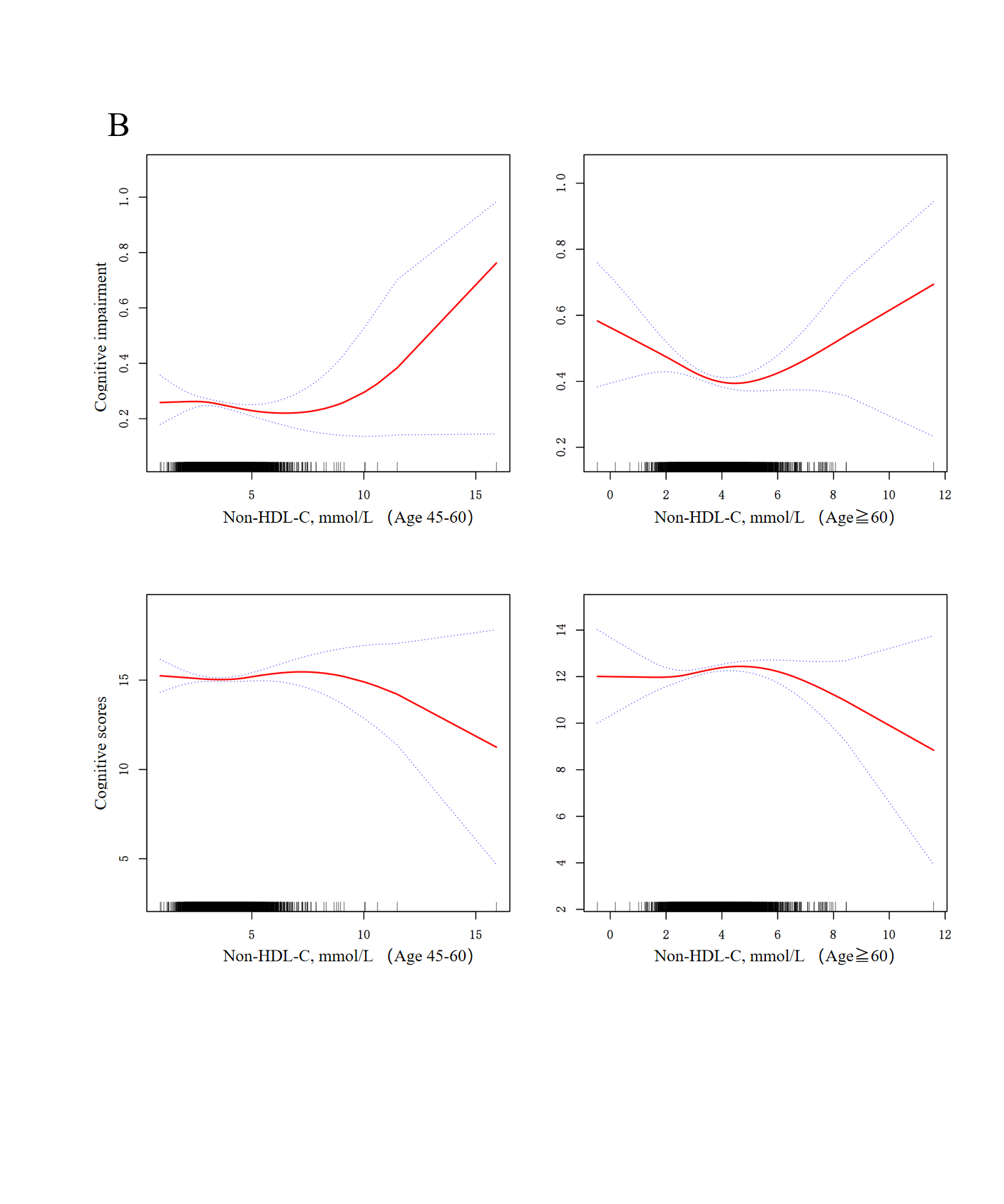


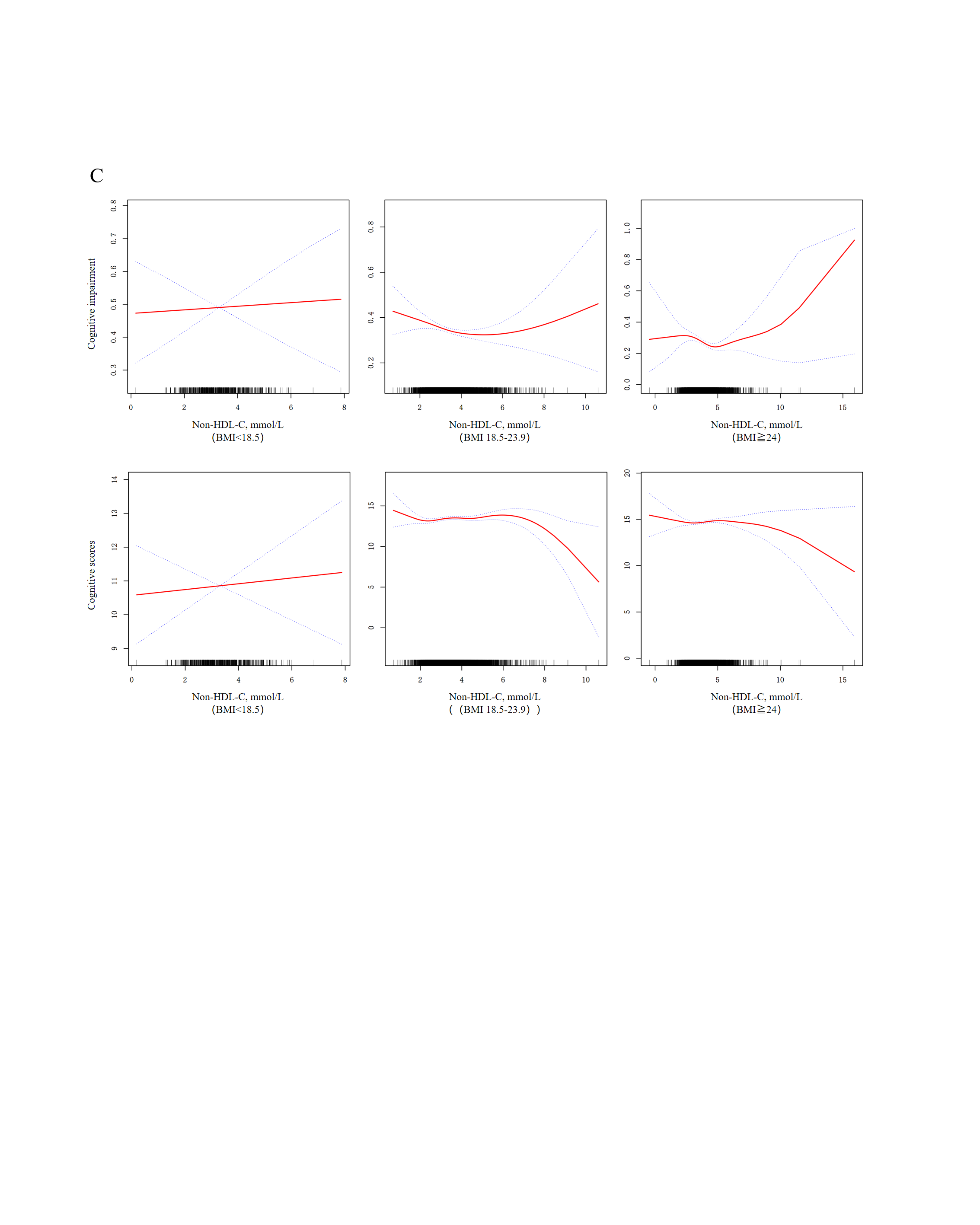


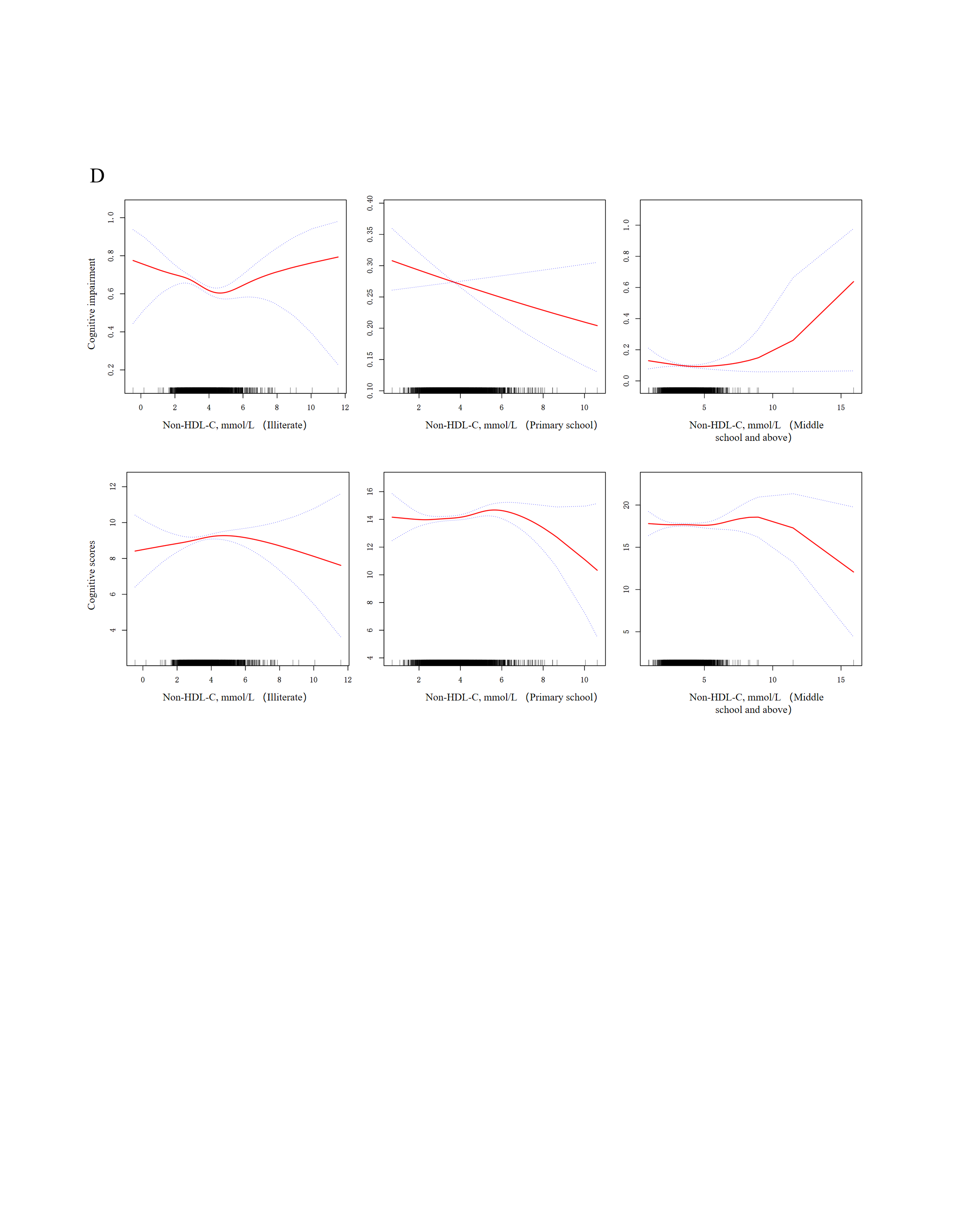


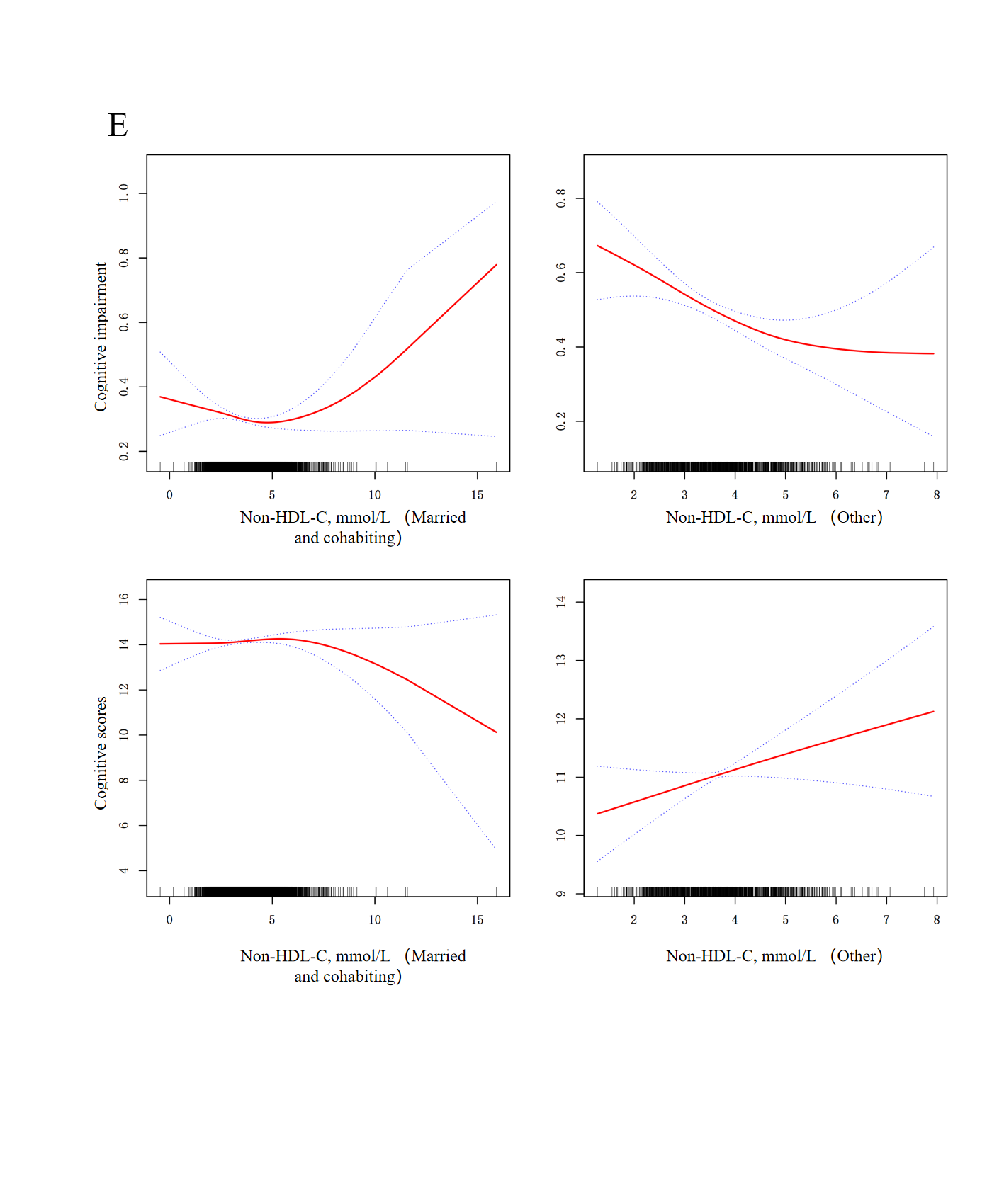


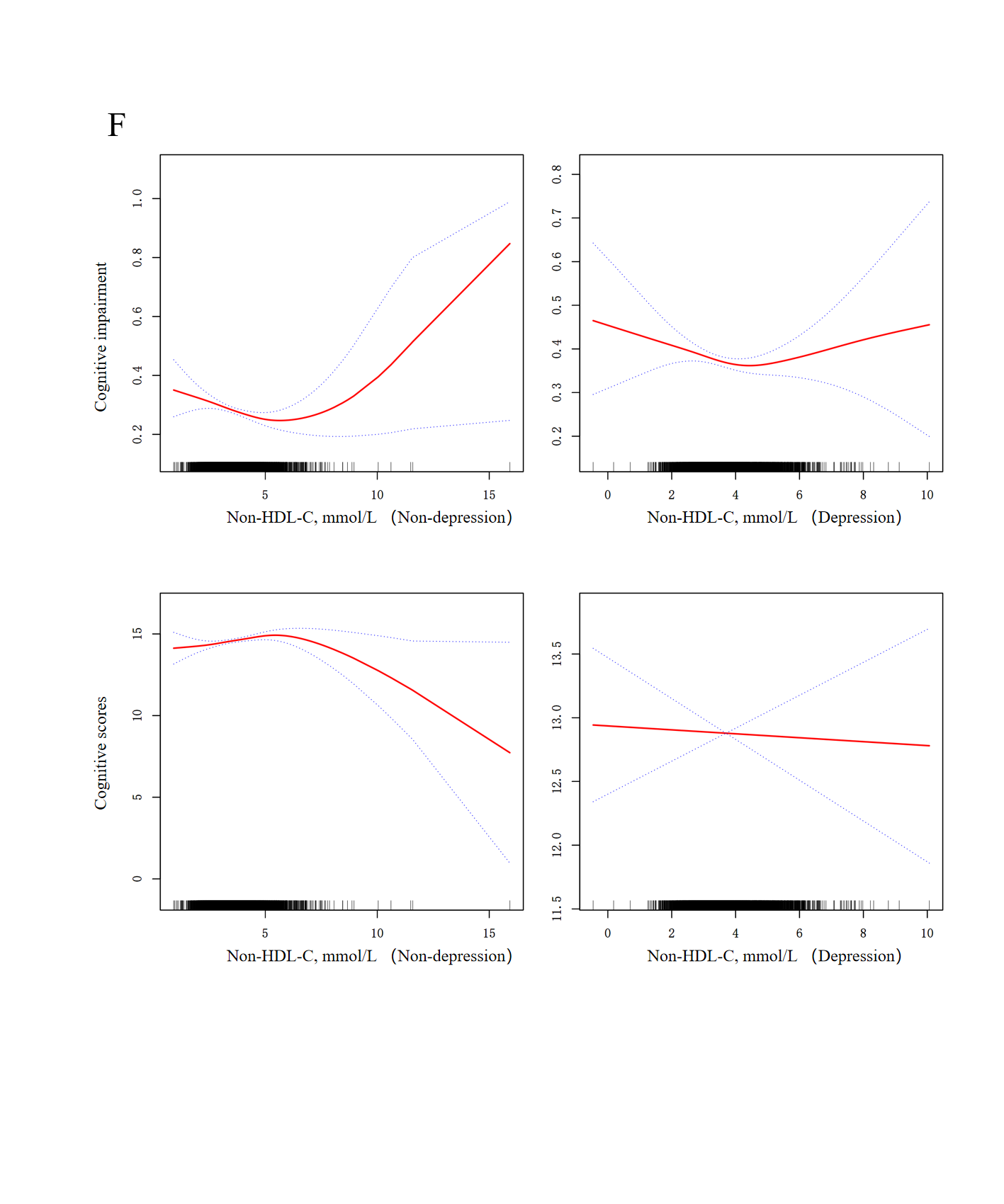


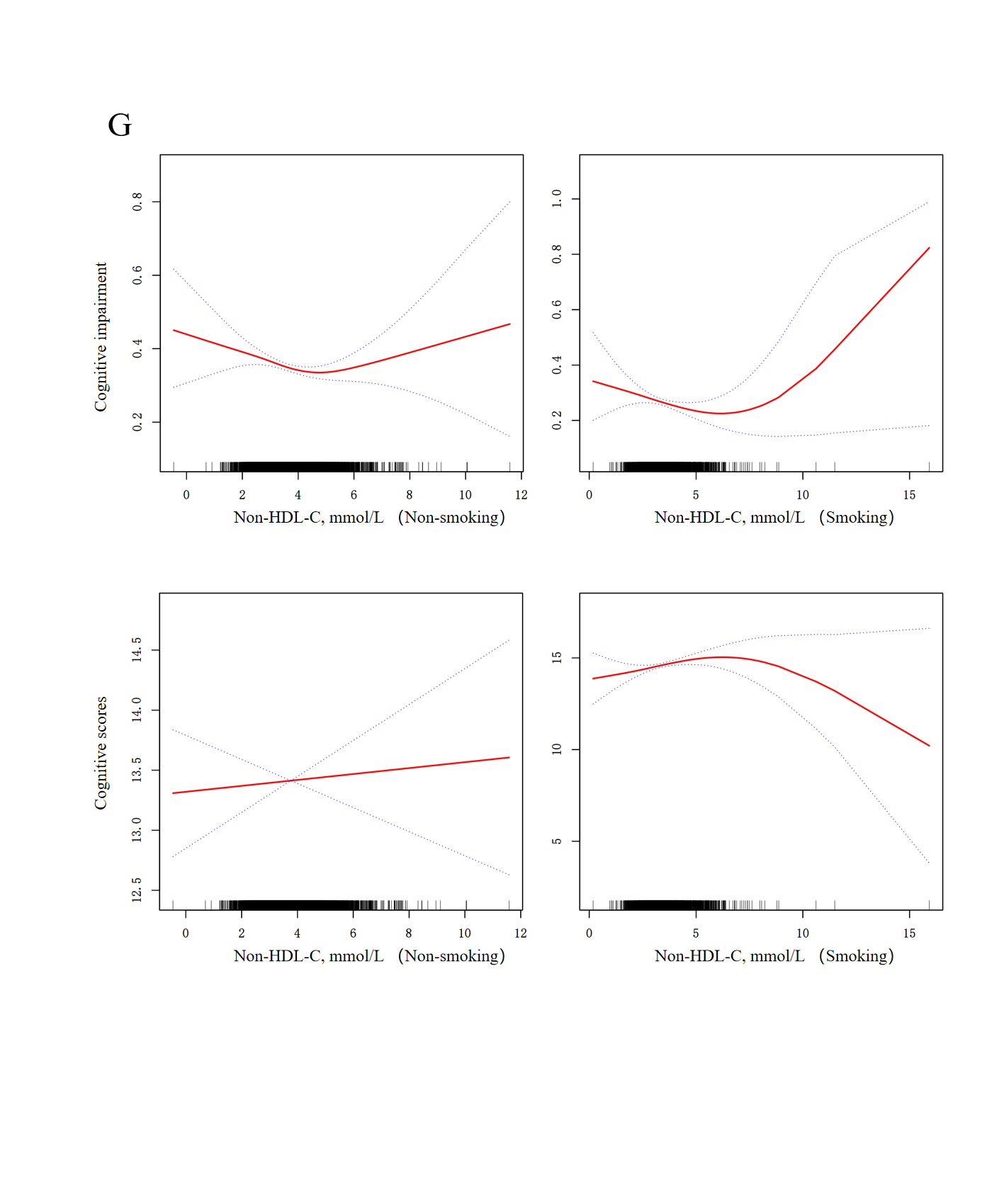


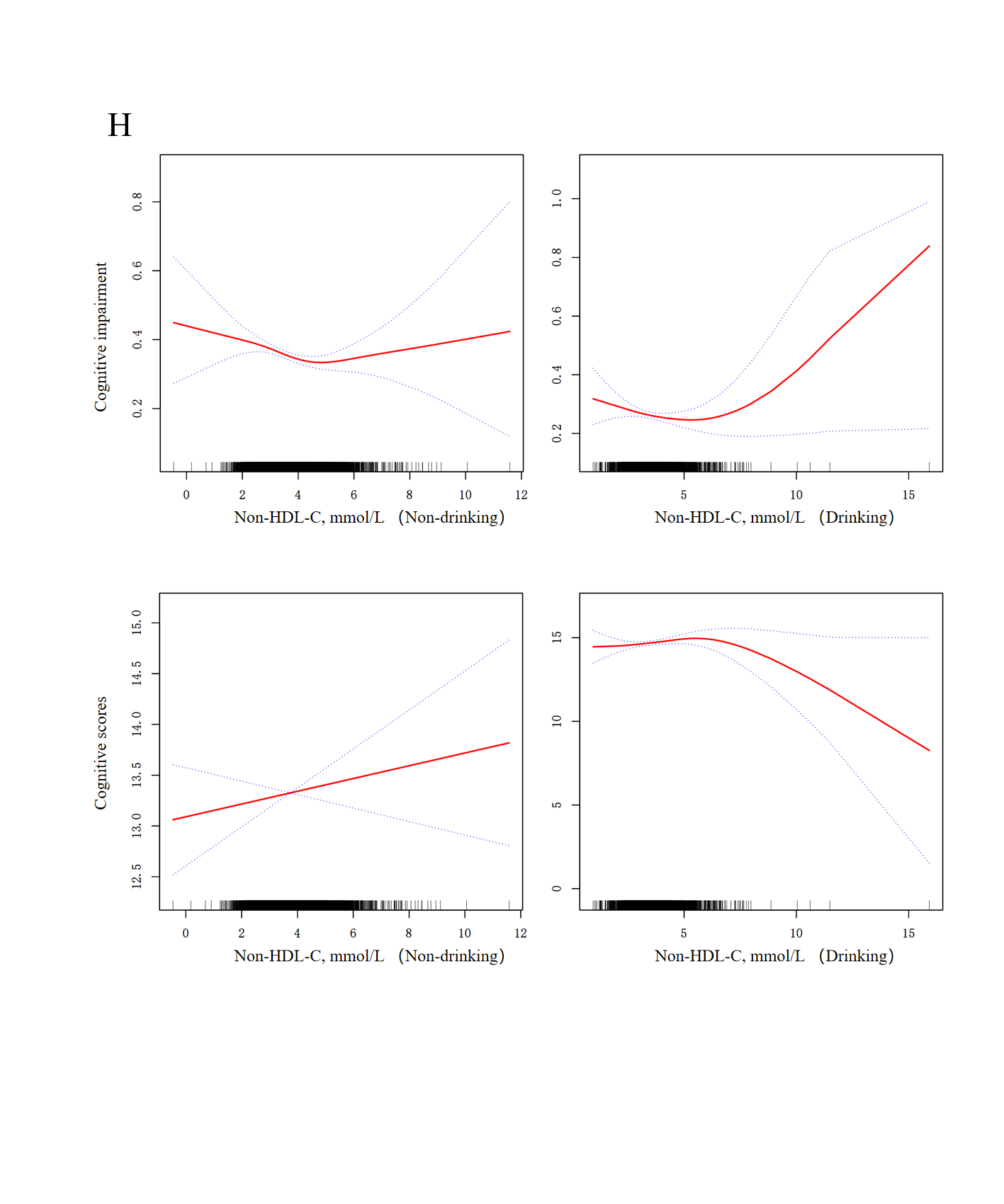


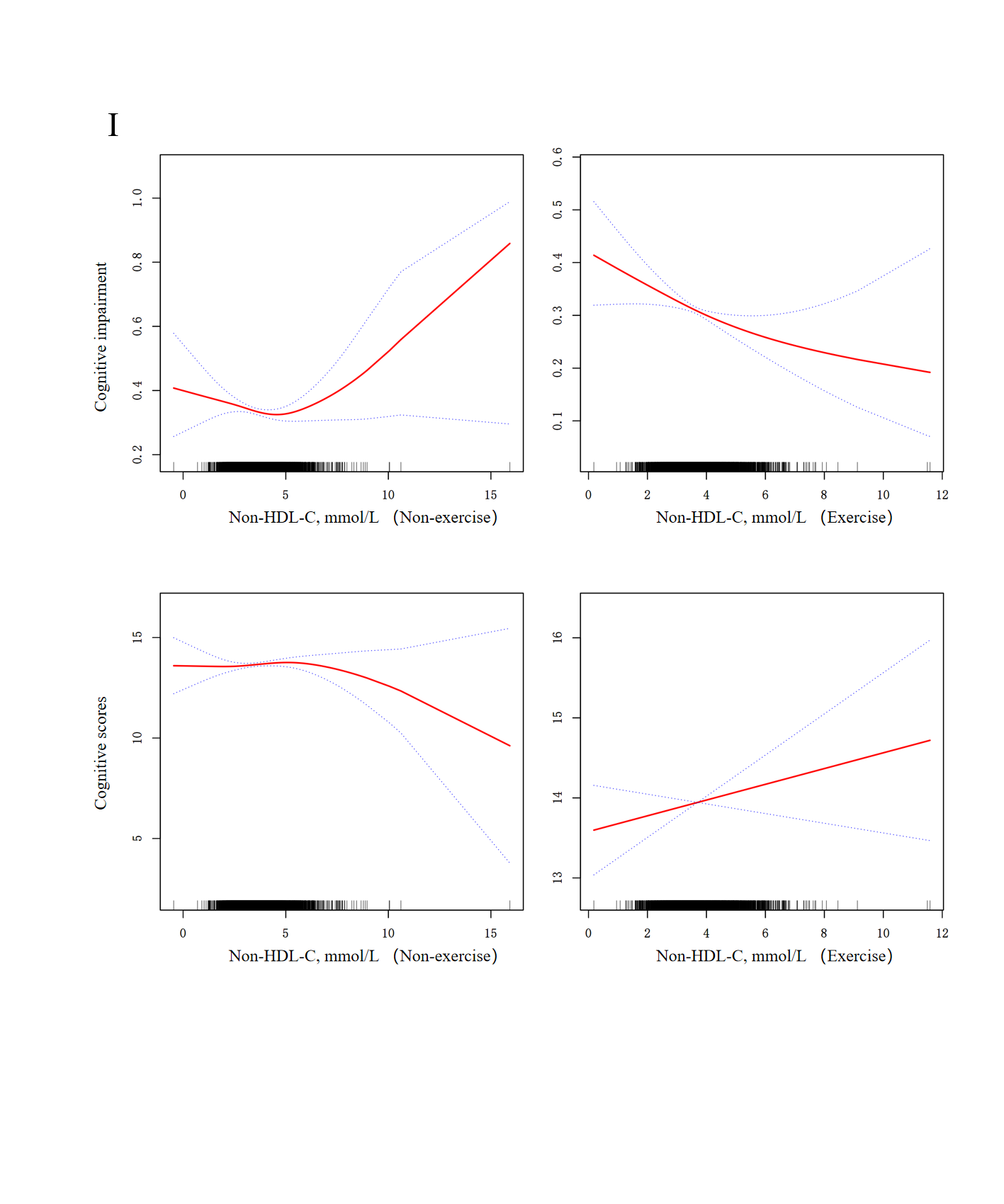


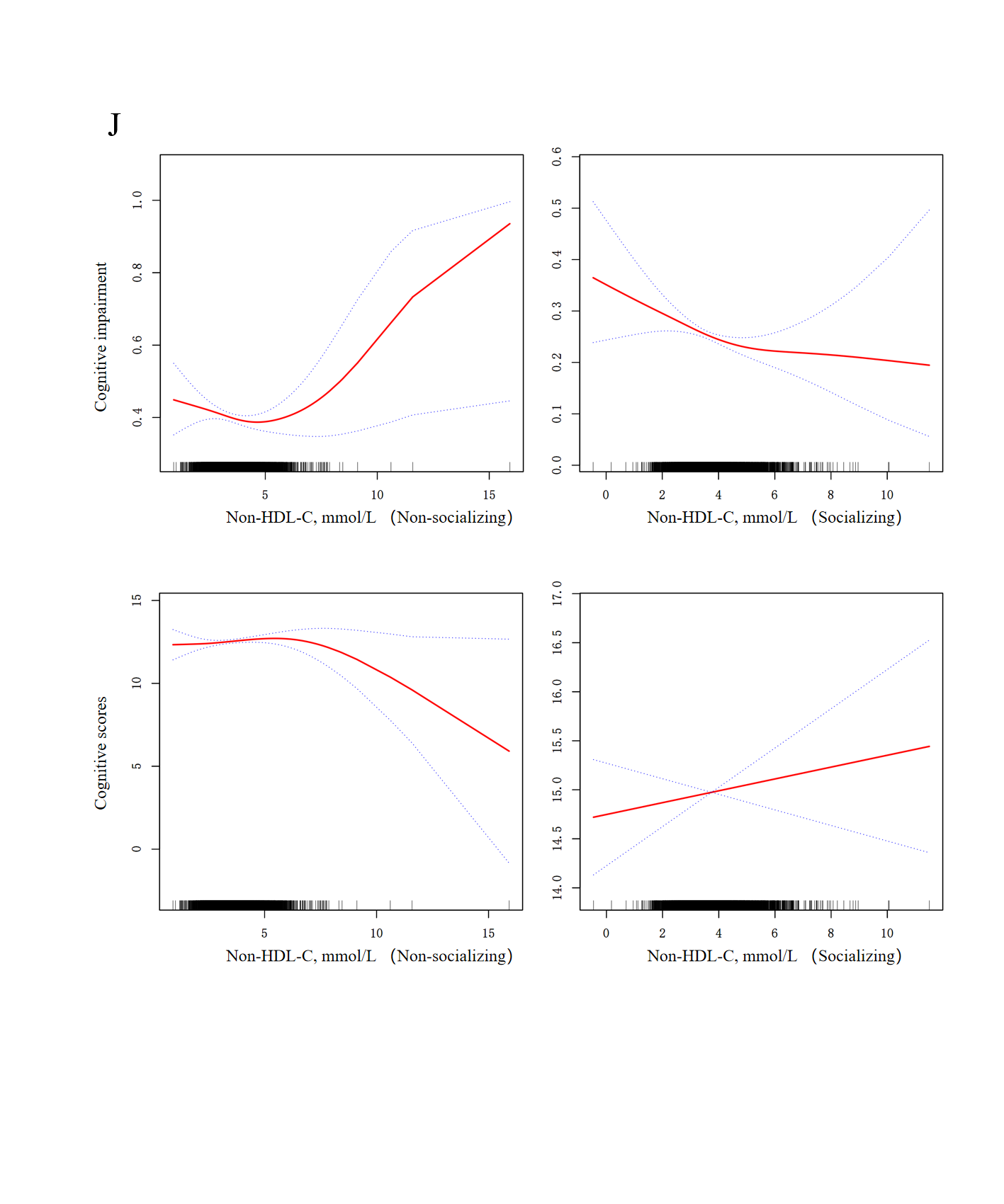

Supplement: Supplementary file 2 — Supplementary Material 2 [file 12889_2024_19164_MOESM2_ESM.docx]
